# Bedside surgery in the newborn infants: survey of the Italian society of pediatric surgery

**DOI:** 10.1186/s13052-020-00889-2

**Published:** 2020-09-16

**Authors:** Gloria Pelizzo, Pietro Bagolan, Francesco Morini, Mariagrazia Aceti, Daniele Alberti, Mario Andermarcher, Luigi Avolio, Fabio Bartoli, Vito Briganti, Sebastiano Cacciaguerra, Francesco S. Camoglio, Pierluca Ceccarelli, Maurizio Cheli, Fabio Chiarenza, Enrico Ciardini, Marcello Cimador, Ennio Clemente, Denis A. Cozzi, Luigi Dall’ Oglio, Ugo De Luca, Carmine Del Rossi, Ciro Esposito, Diego Falchetti, Silvana Federici, Piergiorgio Gamba, Valerio Gentilino, Girolamo Mattioli, Ascanio Martino, Mario Messina, Bruno Noccioli, Alessandro Inserra, Pierluigi Lelli Chiesa, Ernesto Leva, Francesco Licciardi, Paola Midrio, Maria Nobili, Alfonso Papparella, Guglielmo Paradies, Giuseppe Piazza, Alessio Pini Prato, Fabio Rossi, Giovanna Riccipetitoni, Carmelo Romeo, Domenico Salerno, Alessandro Settimi, Jurgen Schleef, Mario Milazzo, Valeria Calcaterra, Mario Lima

**Affiliations:** 1 Department of Paediatric Surgery, Ospedale dei Bambini “V. Buzzi” Children’s Hospital, University of Milano, Milano, Italy; 2 Neonatal Surgery Unit, Department of Medical and Surgical Neonatology, Bambino Gesù Children’s Hospital, IRCCS, Rome, Italy; 3 Azienda Ospedaliera di Cosenza, Cosenza, Italy; 4 Department of Pediatric Surgery, Spedali Civili and University of Brescia, Brescia, Italy; 5 Pediatric Surgery Unit, Ospedale Santa Chiara, Trento, Italy; 6 Pediatric Surgery Unit, Fondazione IRCCS Policlinico S. Matteo, University of Pavia, Pavia, Italy; 7 Pediatric Surgery Unit, University of Foggia, Foggia, Italy; 8 Department of Pediatric Surgery and Urology Unit, San Camillo Forlanini Hospital, Rome, Italy; 9 Deparment of Pediatric Surgery, Ospedale Garibaldi, Catania, Italy; 10 Department of Surgical Sciences, University of Verona, Verona, Italy; 11 Pediatric Surgery Department, Policlinico di Modena, Modena, Italy; 12 Department of Pediatric Surgery, Papa Giovanni XXIII Hospital, Bergamo, Italy; 13 Department of Pediatric Surgery, San Bortolo Hospital, Vicenza, Italy; 14 Pediatric Surgery Unit, Ospedale Santa Chiara, Trento, Italy; 15 Pediatric Urology Unit, Department PRO.MI.SE, University of Palermo, Palermo, Italy; 16 Pediatric Surgery Unit, University of Salerno, Salerno, Italy; 17 Department of Pediatrics, Sapienza University, Rome, Italy; 18 Digestive Endoscopy and Surgery Unit, Bambino Gesu Children’s Hospital-IRCCS, Rome, Italy; 19 Day Surgery Unit, Santobono-Pausilipon Pediatric Hospital, Naples, Italy; 20 Pediatric Surgery Unit, Azienda Ospedaliero-Universitaria di Parma, Parma, Italy; 21 Pediatric Surgery Unit, Federico II Hospital, University of Naples, Naples, Italy; 22 Pediatric Surgery Unit, Niguarda Ca’ Granda Hospital, Milan, Italy; 23 Pediatric Surgery Unit, Infermi Hospital, Rimini, Italy; 24 Department of Pediatric Surgery, University of Padua, Padua, Italy; 25 Unit of Pediatric Surgery, Woman and Child Department, Filippo Del Ponte Hospital - ASST Sette Laghi, Varese, Italy; 26 Department of Pediatric Surgery, G. Gaslini Children’s Hospital, University of Genoa, Genoa, Italy; 27 Pediatric Surgery Unit, Salesi Children’s Hospital, Politecnico delle Marche University, Ancona, Italy; 28 Division of Pediatric Surgery, Department of Medical Sciences, Surgery and Neuroscience, University of Siena, Siena, Italy; 29 Department of Neonatal and Emergency Surgery, Meyer Children’s Hospital, Florence, Italy; 30 Surgical Oncology Unit, Department of Surgery, IRCCS Bambino Gesù Children’s Hospital, Rome, Italy; 31 Pediatric Unit, Santo Spirito Hospital, University of Chieti-Pescara, Pescara, Italy; 32 Department of Pediatric Surgery, Fondazione IRCCS Ca’ Granda, Ospedale Maggiore Policlinico, University of Milan, Milan, Italy; 33 Pediatric Surgery Unit, Presidio Ospedaliero CTO, Iglesias, Italy; 34 Pediatric Surgery, Ca’ Foncello Hospital, Treviso, Italy; 35 Department of Woman, Child and General and Specialized Surgery, University of Campania “Luigi Vanvitelli”, Naples, Italy; 36 Pediatric Surgery Unit, Ospedale Giovanni XXIII, Bari, Italy; 37 Pediatric Surgery Unit, Sant’Antonio Abate Hospital, Trapani, Italy; 38 Unit of Pediatric Surgery, The Children Hospital, Azienda Ospedaliera SS Antonio e Biagio e Cesare Arrigo, Alessandria, Italy; 39 Pediatric Surgery Unit, Azienda Ospedaliero-Universitaria Maggiore della Carità , Novara, Italy; 40 Department of Human Pathology of Adult and Childhood “Gaetano Barresi”, Unit of Pediatric Surgery, University of Messina, Messina, Italy; 41 Pediatric Surgery Unit, Azienda Ospedaliera Pugliese-Ciaccio, Catanzaro, Italy; 42 Pediatric Surgery Unit, Federico II Hospital, University of Naples , Naples, Italy; 43 Department of Pediatric Surgery, Institute for Maternal and Child Health - IRCCS Burlo Garofolo, Trieste, Italy; 44 Pediatric Surgery Unit, Ospedale del Bambini “G. Di Cristina”, ARNAS Civico-Di Cristina-Benfratelli, Palermo, Italy; 45 Pediatric and Adolescent Unit, Department of Internal Medicine, University of Pavia and Pediatric Unit V. Buzzi Children’s Hospital, Milan, Italy; 46 Department of Pediatric Surgery, University of Bologna, Bologna, Italy

**Keywords:** Neonatal intensive care unit, Bedside surgery, Operative room, Intrahospital transport, Critically ill neonates

## Abstract

**Introduction:**

This is the report of the first official survey from the Italian Society of Pediatric Surgery (ISPS) to appraise the distribution and organization of bedside surgery in the neonatal intensive care units (NICU) in Italy.

**Methods:**

A questionnaire requesting general data, staff data and workload data of the centers was developed and sent by means of an online cloud-based software instrument to all Italian pediatric surgery Units.

**Results:**

The survey was answered by 34 (65%) out of 52 centers. NICU bedside surgery is reported in 81.8% of the pediatric surgery centers. A lower prevalence of bedside surgical practice in the NICU was reported for Southern Italy and the islands than for Northern Italy and Central Italy (Southern <Northern<Central, *p* < 0.03). The most frequent clinical characteristics of neonates was preterm neonates with birthweight < 1200 g, with cardiorespiratory instability and/or ventilatory dependence. The most frequently selected indications to surgery were pneumothorax, pleural effusion, pericardial effusion, central venous catheter (CVC) positioning, intestinal perforation, patent ductus arteriosus ligation and congenital diaphragmatic hernia. More than 60% of respondents report no institutional recommendations and dedicated informed consent on bedside surgical procedures. The lack of dedicated areas and infrastructures is considered a relative contraindication to the performance of bedside surgery.

**Conclusion:**

Bedside surgery is performed in the majority of the Italian pediatric surgery centers included in this census. The introduction of a national set of surgery guidelines would be widely welcomed.

## Introduction

Critically ill neonates in the neonatal intensive care unit (NICU) are often in need of surgical interventions [1, 2]. Patients are usually transferred to an operating room (OR) outside of the NICU by intrahospital transport [3–5]. Transportation of critical patients is associated with a high risk of aggravating their clinical status and increased complications rate, up to 70% [4–7]. To avoid the transport of critically ill newborns, some Institutions have launched NICU OR programs in recent years [1, 2, 8–11].

As reported, candidates for NICU bedside surgery are the more unstable neonates on high-frequency oscillatory ventilation, inhaled nitric oxide therapy, or on extracorporeal membrane oxygenation (ECMO) [1, 12, 13] Importantly, NICU bedside surgery also provides continuity of care with the same intensive care team. The benefits and risks of performing surgery on critically ill newborns in the OR within the NICU compared to those of conducting surgery outside the NICU have already been reported in the literature, including the maintenance of the cardiovascular stability, the decreased risk of recurrent accidents during transportation such as hypothermia and dislocation of vascular accesses or endotracheal tubes [1, 9, 13–15].

At the same time, there are as yet no definite guidelines for the planning of bedside surgery: these would be useful for, amongst other things, coordination of the multidisciplinary team and assessment of the facility, including procedures for the transport of personnel and the available equipment. In Italy, as in most countries in Europe, no data are available on the distribution and organization of pediatric surgical institutions that perform NICU bedside surgery.

As a preliminary step towards the standardization of national NICU bedside surgery, the Italian Society of Pediatric Surgery (ISPS) conducted the first official census of the Italian pediatric surgery centers to appraise bedside surgical activities in newborn infants. Organizational characteristics enabling bedside practice are evaluated and reported here.

## Methods

The survey was conducted in three operative steps:

- *First step:* preparation of a complete list of the Italian pediatric surgery centers with e-mail contacts. Pediatric surgery center was defined as a formally recognized structure in a public hospital, belonging either to the national health service or to a university, with beds and staff specifically dedicated to the care of pediatric surgical patients. Structures in private for-profit hospitals and pediatric surgical beds in general surgery and pediatrics departments were excluded.

Italy was divided into three geographical areas each encompassing several political regions: Northern (Piemonte, Valle d’Aosta, Liguria, Lombardia, Emilia-Romagna, Veneto, Friuli-Venezia Giulia and Trentino-Alto Adige), Central (Lazio, Marche, Toscana and Umbria), Southern and islands (Abruzzo, Molise, Campania, Puglia, Basilicata, Calabria, Sicilia and Sardegna)

- *Second step:* preparation of the survey questionnaire (Table 1), designed after thorough consultation among ISPS executive board members. The aim was to produce a tool that was simple and rapid to complete, but that would collect as many useful details as possible (general data, staff data and workload data of the centers). The questionnaire was distributed by means of an online cloud-based software instrument (Survey Monkey) to all pediatric centers.
- *Third step:* collection and processing of data obtained with the questionnaire.

**Table 1:** Questionnaire and responses of the all responder centers

Questions	Choices	Answer (Total ***n*** = 34) n (%)
The institution in which you work is provided with:	□ Neonatal Intensive Care Unit	33 (97.06)
	□ Pediatric Surgery Unit	34 (100)
	□ Prenatal diagnosis and counseling Unit	27 (79.41)
The institution where you work is located:	□ Northern	15 (44.12)
	□ Southern and islands	14 (41.18)
	□ Central	5 (14.71)
How many doctors work in your operative unit?	□ ≤3	1 (2.94)
	□ 4	3 (8.82)
	□ 5	4 (11.76)
	□ 6–10	19 (55.88)
	□ > 10	7 (20.59)
How many doctors are dedicated to neonatal surgery in your unit?	□ 1	2 (2.88)
	□ 2	4 (11.78)
	□ 3	6 (17.65)
	□ 4	7 (20.59)
	□ 5	6 (17.65)
	□ > 5	9 (26.47)
How many nurses work in your unit?	□ 5	2 (5.88)
	□ 6–10	5 (14.71)
	□ 11–15	13 (38.24)
	□ 16–20	7 (20.59)
	□ > 20	7 (20.59)
How many nurses are dedicated to neonatal surgery in your unit?	□ < 5	20 (62.50)
	□ 5–10	2 (6.32)
	□ 11–15	7 (21.88)
	□ > 15	3 (9.38)
How many beds are there in your pediatric surgery unit?	□ ≤10	9 (26.47)
	□ 11–15	10 (29.41)
	□ 16–20	9 (26.47)
	□ > 20	6 (17.65)
**Practices on surgery**		
How many beds are dedicated to neonatal surgery in NICU and/or in your surgical unit?	□ < 5	25 (75.76)
	□ 5–10	6 (18.18)
	□ 11–15	1 (3.03)
	□ > 15	1 (3.03)
How many neonatal surgical interventions in operative rooom or bedside are performed annually (last year) in your surgical unit?	□ < 20	7 (20.59)
	□ 21–30	15 (44.12)
	□ 31–50	6 (17.65)
	□ > 50	6 (17.65)
Is bedside surgery routine in selected unstable cases in your NICU?	□ Yes	27 (79.42)
	□ No	7 (20.59)
What do you think are the main problems that could contraindicate a surgical intervention in the NICU on selected patients?	□ Unavailability of rooms at the NICU	23 (74.19)
	□ Lack of legal regulation	11 (35.48)
	□ Increased technical risk due to limited surgeon experience	11 (35.48)
	□ Increased infection risk	9 (29.03)
	□ Unavailability of the neonatological team to consider this approach	9 (29.03)
	□ Denial of Head of Health Management Unit	2 (6.45)
	□ Other	5 (16.13)

### Statistical analysis

All analyses were performed using Stata 15 (StataCorp, College Station, TX, USA). Categorical data were expressed as numbers and percentages. The Fisher exact test was used to assess differences in distribution of categorical variables.

## Results

A total of 34 out of 52 centers answered the questionnaire. All the data collected are reported in Table 1.

### General and staff data for surveyed pediatric surgery centers

The geographical distribution of the centers who responded to the survey is: Northern 15/34 (44%), 5/34 Central (15%) and Southern and islands 14/34 (41%) (Table 1).

NICU are present in 33/34 (97%) of respondent centers. Most report 11–15 beds for the pediatric surgery unit and < 5 beds for the surgical neonate. Urgent/emergent surgery can be performed in 97% of the institutions. Prenatal diagnosis and counseling facilities are also reported in 27/34 (79%) of the centers (Table 1).

The majority of centers (64%) report less than 30 neonatal surgeries /last year (bedside+operative room).

Three quarters of the centers (76%) have 6 or more pediatric surgeons working in the pediatric surgery unit. In all centers at least 1 surgeon is dedicated to the surgical neonate, while 9 (26%) have more than 5 surgeons dedicated to the surgical neonate (Table 1).

Between 11 and 15 nurses work in the pediatric surgery unit in 13/34 (38%) centers, with fewer than 5 nurses dedicated to neonatal nursing in 20/34 (63%) (Table 1).

### Bedside surgery in the NICU: workload data

**Fig. 1:**
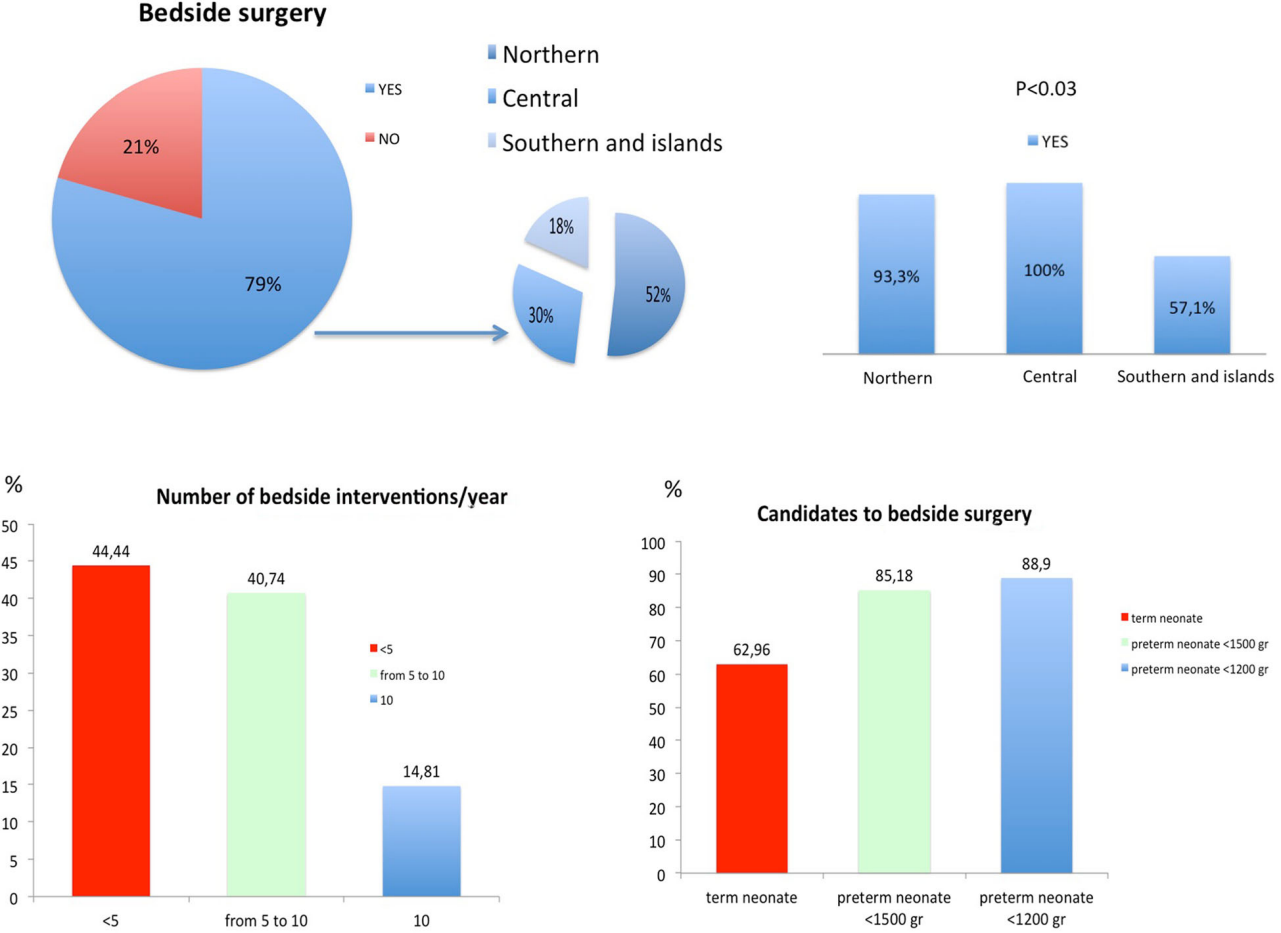
Bedside surgery in NICUs: prevalence and workload

Bedside surgery is reported to be performed in 27/33 (81.8%) of the centers that have a NICU. Of the 27, 14 (52%) are in Northern Italy, 5 (18%) in Central Italy and 8 (30%) in Southern Italy and islands, with a lower prevalence (*p* < 0.03) of bedside practice in Southern Italy and the islands (8 of the 14 respondent centers) compared to Northern (14 of the 15 respondent centers) and Central (5 of the 5 respondent centers) Italy (Table 1, Fig. 1). The lack of a dedicated area and infrastructures are described as the main relative contraindication to bedside surgery (74%), Table 1.

In the centers with bedside practice, there were < 5 bedside surgical interventions per year in 44% of the centers, from 5 to 10 per year in 41% and > 10 per year in 15%. No bedside surgical interventions are performed via thoracic or laparoscopic approach (Table 2).

**Table 2:** Questionnaire and responses othe the centers with bedside practice

Questions	Choices	Answer (Total ***n*** = 27) n (%)
**Practices on surgery**		
2002How many newborns per year are treated with surgery in NICU?	□ < 5	12 (44.44)
	□ 5–10	11 (40.74)
	□ > 10	4 (14.81)
Does the institute where you work receive babies from other centers for urgent surgical treatment?	□ Yes	27 (100)
Is the NICU, or part of the premises of which it is constituted, equipped with positive pressure?	□ Yes	19 (70.37)
	□ No	8 (29.62)
**Bedside selection criteria**		
Which patients are most likely to be considered for surgical interventions in NICU according to weight and gestational age?	□ Preterm neonates with birthweight < 1200 g	24 (88.89)
	□ Preterm neonates with birthweight < 1500 g	23 (85.18)
	□ Term neonates	17 (62.96)
What are the general criteria for selecting candidates for surgery in NICU?	□ Cardiorespiratory instability	27 (100)
	□ Ventilatory dependance	17 (62.96)
	□ Weight	17 (62.96)
	□ Gestational age	13 (48.14)
	□ Other	6 (22.22)
What are the procedures and pathologies that may be considered as indications to surgery in NICU?	□ Pneumothorax drenage	25 (92.60)
	□ Pneumoperitoneum treatment	24 (88.89)
	□ Pleural effusion drenage	23 (85.18)
	□ Pericardial effusion drenage	23 (85.18)
	□ CVC positioning	22 (81.48)
	□ Patent ductus arteriosus ligation	18 (66.67)
	□ Congenital diaphragmatic hernia repair	18 (66.67)
	□ NEC treatment	17 (62.96)
	□ Dialysis catheter positioning	14 (51.85)
	□ Thoracic surgery	2 (7.40)
	□ Other	2 (7.40)
Is bedside surgery performed via thoracic or laparoscopic approach in your unit?	□ No	27 (100)
**Practices on perioperative monitoring**		
What kind of anesthetic procedure is implemented at surgery in the NICU?	□ Endovenous	25 (92.60)
	□ Inhaled	16 (59.25)
	□ Locoregional	12 (44.45)
Who monitors the newborn during the surgical procedure?	□ Neonatologist	5 (18.51)
	□ Anesthesiologist	2 (7.40)
	□ Both	20 (74.07)
Who manages sedation and pain control procedures after surgery?	□ Multidisciplinary management	21 (77.78)
	□ Neonatologist	5 (18.51)
	□ Pediatric surgeon	4 (14.81)
	□ Anesthesiologist	2 (7.40)
**Organizative procedures**		
Is there a specific reference to surgery being conducted at the bedside in the informed consent?	□ Yes	9 (33.33)
	□ No	18 (66.67)
Is there an official pathway or institutional protocol for surgery in the NICU?	□ Yes	8 (29.62)
	□ No	19 (70.37)
Do you refer to specific literature sources to support the practice of NICU surgical procedures?	□ Yes	15 (55.56)
	□ No	12 (44.44)
Do you consider it important, for the purpose of quality of assistance in this fragile category, to reach a uniform vision on the appropriacy of proceeding with selected surgical procedures within the NICU?	□ Yes	25 (92.60)
	□ No	2 (7.40)

In 89% of centers, preterm neonates with birthweight < 1200 g are the category of babies most likely to undergo NICU bedside surgery (Fig. 1, Table 2). In all Institutions, cardiorespiratory instability (100%) and ventilator dependence (63%) are the most-reported criteria in the selection of patients (Table 2).

Pneumothorax drenage (92%), intestinal perforation (89%), pleural effusion drenage (85%), pericardial effusion drenage (85%), central venous catheter (CVC) positioning (81%), patent ductus arteriosus ligation (67%) and congenital diaphragmatic hernia repair (67%) are considered indications to bedside surgery (Table 2).

Intravenous general anesthesia is the most frequently performed anesthesia (93%) although also inhaled (59%) and locoregional (44%) anesthesia are performed in the NICU setting. Multidisciplinary management of during-surgery and post-surgery pain are widely reported (Table 2).

There were no institutional recommendations on bedside surgical procedures are available in 19/27 (70%) of the centers. In all centers general written consent for surgery was obtained, but in 18/27 (67%) no dedicated informed consent for bedside was available.

Of the respondent centers, 94% consider necessary drafting a national NICU bedside surgery guideline.

## Discussion

This is the first report, as far as we are aware, of the geographical distribution and workload of Italian pediatric surgical institutions where bedside surgery is performed in the NICU. In Italy, bedside surgery in the NICU is widely practiced and is performed in more than 79% of the respondent centers, although we found some regional differences.

Even if the number of procedures were not recorded, indications to bedside surgery were in line with those reported in the literature, and the bedside approach is adopted for several procedures in the NICUs surveyed including open abdominal surgery (necrotizing enterocolitis, intestinal perforation, abdominal wall defect repair/reduction, stoma creation), and thoracic surgery (congenital diaphragmatic hernia, tracheostomy, drainage), central line placement, cardiac surgery (ligation of patent ductus arteriosus) [1, 2, 9, 16–21] Neonates in need of surgery are traditionally transferred to the main OR, outside the NICU. Most of them are premature with a low birth weight, cardiovascular instability and prolonged ventilator support. The transport of unstable neonates to and from the OR is associated with significant morbidity that may adversely affect outcomes in compromised patients, despite improvements in intrahospital transportation, equipment and experience [1, 3]. Duration of transportation and the severity of the patients’ symptoms are also crucial factors affecting complications [4, 5]. Recurrent accidents include hypothermia, change in variations in heart rate and blood pressure, and dislocation of vascular accesses or endotracheal tubes [3–7]. Bedside surgery in the NICU may avoid accidents during transport, especially for critical and unstable neonates on high-frequency oscillatory ventilation, inhaled nitric oxide therapy, or even ECMO [1, 12, 13]. Surgery in the NICU provides continuity of care by the same intensive care team and guarantees the best care [1]. Therefore, every neonatal ICU planner should create infrastructures for bedside surgery to improve the safety of care [2]. The heterogeneity of the NICU bedside surgery situation in Italy suggested by present survey calls for efforts to regulate the practice in order to obtain the optimal the standard of care in the whole country.

In the neonatal patient, surgery requires monitoring of perfusion throughout the operation. In particular, monitoring of brain perfusion is key to improving the survival of these fragile neonates because of the hypoxic ischemic injury risk due to stress and prematurity. For this reason, for bedside NICU surgery to be possible, a dedicated area with infrastructures like central oxygen, suction, compressed air and multiparamonitors is mandatory. In addition, an increased risk of infections following bedside surgery has been reported [9, 14] in case of NICU not provided of a dedicated area for surgery. It is possible that the lack of these facilities found in present survey may represent a major impediment to the spread of bedside surgical procedures in Italy.

Our survey indicates that there are few dedicated teams of surgeons and nurses in Italian centers. All invasive procedures involved the pediatric surgeon advice and multidisciplinary management is widely adopted. According to the British Association of Perinatal Medicine guidelines ‘Standards for Hospitals Providing Neonatal Intensive and High Dependency Care and Categories of Babies requiring Neonatal Care’, level III Units should provide the whole range of neonatal medical care but not necessarily to all the specialist services [22] such as bedside neonatal surgery. Where this is available, a team should typically consist of a senior neonatal surgeon, two neonatal surgeons as assistants (one may be a trainee), two trained surgical nurses (one scrub nurse and the other a floor nurse), one technician to maintain the instruments and two neonatal anesthetists. In addition, a neonatologist should attend the surgery to support the anesthetist in continuous monitoring of the patient during surgery and to adjust ventilation parameters as required by the patient’s conditions. At the same time, the regular activity of the NICU must not be disrupted by surgery [1]. A NICU dedicated surgical team enables optimal reach and utilization of resources, but solutions for optimizing children’s surgical care remain under debate worldwide [23, 24].

So far there are no Italian recommendations for bedside surgery in the NICU, and more than 50% of the centers do not consult specialist literature sources to support the practice of NICU surgical intervention. In general, because the feasibility and safety of NICU bedside surgery are well documented [8–15], and the Lancet Commission on Global Surgery [24] on surgical care encourages the introduction of this new therapeutic approach to address the needs of children, no special permission is required. The results of this Survey may be used to optimize the organization of infrastructure, service delivery, training and research, however the development of specific National guidelines may help in the national spread and standardization of NICU bedside surgery. Such guidelines should include an optimal National resources document outlining the personnel, equipment, facilities, procedures, training, research and quality improvement components necessary at all levels of care [24]. Additionally, a surgical safety checklist could be adopted to improve teamwork, communication and adherence to procedural steps and also as a useful learning tool to help junior doctors perform invasive procedures in the NICU [1, 2].

## Conclusions

Bedside surgery is performed in the majority of the Italian pediatric surgery centers included in this census. The introduction of a national set of surgery guidelines, a formal protocol for comprehensive perioperative planning, a dedicated surgical safety checklist and informed consent would be widely welcomed.

## Data Availability

All data generated or analysed during this study are included in this published article.

## Abbreviations

CVC: Central venous catheter
ISPS: Italian Society of Pediatric Surgery
NICU: Neonatal intensive care unit
OR: Operative room
